# Injuries to Users of Single-Track Vehicles

**DOI:** 10.3390/ijerph191912112

**Published:** 2022-09-24

**Authors:** Piotr Konrad Leszczyński, Justyna Kalinowska, Krzysztof Mitura, Daryna Sholokhova

**Affiliations:** 1 Faculty of Medical Sciences and Health Sciences, Siedlce University of Natural Sciences and Humanities, ul. Bolesława Prusa 14, 08-110 Siedlce, Poland; 2 Emergency Medical Service, ul. Dr Jana Huberta 37, 05-300 Mińsk Mazowiecki, Poland

**Keywords:** injuries, single-track vehicles, trauma, bicycles, motorcycles, scooters

## Abstract

Introduction: Single-track vehicles (including, among others, scooters, bicycles, mopeds, and motorcycles) are becoming increasingly popular means of transport, especially in large cities. A significant disadvantage of single-track vehicles is the low level of protection of users’ bodies during road accidents, which causes life-threatening injuries. The aim of this study is to characterize the injuries of users of single-track vehicles. Material and methods: An analysis of medical documentation of the ambulance service in the region of central Poland covered cases in 2019–2020. Out of 17,446 interventions, a group of 248 road incidents involving single-track vehicles was selected. The data included the scene of the event, the sociodemographic data of the casualties, the injuries suffered, and the clinical diagnoses. Analyses of the correlation of variables with the chi-squared and Spearman’s Rho tests were applied. All results were considered significant at *p* < 0.05. Results: In the analyzed period, trips of men accounted for 83.5% of all of the interventions (n = 207), while trips of women accounted for 16.5% (n = 41). The mean age of the victims was 45.66 years (SD ± 20.45). Taking into account the division of single-track vehicles, individual cases were recorded with the participation of bicycles (n = 183), motorcycles (n = 61), and scooters (n = 4). Taking into account the type of event, the following were distinguished: deductions (n = 62), falls (n = 179), and sickness (n = 7). The most common injuries were to the heads of cyclists (n = 101, which constitutes 55.19% of all injuries), lower limb injuries in motorcyclists (n = 35; 57.38%), and head injuries in scooter users (n = 3; 75%). The locations of sustained injuries significantly correlated with the type of vehicle in the cases of head injuries (*p* = 0.046), spine/back injuries (*p* = 0.001), pelvis injuries (*p* = 0.021), and lower limb injuries (*p* = 0.001). Conclusions: The users of single-track vehicles injured in road accidents were more often men than women. The characteristics of the injuries depended on the type of vehicle. The lack of adequate body protection significantly increases the likelihood of death or damage to health. It is advisable to promote safety rules among users of single-track vehicles, with a particular emphasis on the protection of individual parts of the body.

## 1. Introduction

The popularity of single-track vehicles as means of transport has increased significantly nowadays. More and more often on the streets one can observe people using single-track vehicles, and this is especially noticeable in large cities. Single-track vehicles include vehicles in which the wheels are aligned (e.g., motorcycles, mopeds, bicycles, or scooters). Despite their many advantages, such as finding a faster alternative to move around streets with heavy city traffic, single-track vehicles also have many disadvantages. One of the most significant disadvantages of this type of vehicle is the lack of adequate protection and safety in accidents, which is not the case for motor vehicles. The safety of a person riding a single-track vehicle mainly depends on their use of protective clothing. The use of a helmet, pads, boots, gloves, a reinforced jacket, and pants greatly improves the level of road safety [1,2].

Traffic accidents, in which the injured are single-track vehicle users, often occur as a result of a lack of technical efficiency or appropriate equipment of the vehicle, lack of competence and knowledge of the driver of a single-track vehicle (e.g., failure to adapt the appropriate speed to weather conditions or the situation on the road), as well as inattention on the part of the drivers of other vehicles. As a result of traffic accidents, the injured may suffer injuries posing a threat to their life and health, requiring emergency measures [3,4]. 

**Literature review**: Incidents involving single-track vehicles are difficult to assess. Scooters and bicycles usually lack ride recorders and speedometers. The force of an impact is easier to measure via damage to a car than via damage to a bicycle. As demonstrated in the work by Spörri E. et al. (2021), it is important to determine the speed and track for a reliable assessment of the risk of death [5]. Modern technological solutions provide users with e-bikes and e-scooters (electric vehicles) to reach higher speeds, which carries serious risks. However, Hu L. et al. (2020) have shown that the overall pattern of injuries is similar between electric and regular bicycle users [6]. An analysis of simulated events by Fenca R. et al. (2022) draws attention to elements of single-track vehicle frames, which can also cause serious injuries during collisions [7]. The lack of cockpits covering users’ bodies, as well as the lack of protection (e.g., helmets), significantly increases the risk of head injuries in cyclists compared to motorcyclists, which was demonstrated by the study by Kent T. et al. (2022) [8]. Bascher D. et al. (2019) additionally confirmed that the speed achieved by e-bike users significantly increases the severity of craniocerebral injuries [9]. Data in the literature on specific injuries that correlate with types of vehicles are still insufficient. The statistics kept by the police may be incomplete [10]; therefore, data from medical units should be considered as a reliable source of information.

**Aim:** In the conducted study, an attempt was made to characterize road incidents in which the victims were single-track vehicle users. The main aim of this study was to characterize the injuries of users of single-track vehicles. The specific objectives included the following:

(1) Correlation of injuries with types of vehicles;
(2) Creating a profile of a patient involved in an accident on a two-wheeler;
(3) Specification of the characteristics of events.

## 2. Material and Methods

A retrospective study was carried out on the ambulance service in central Poland, covering medical documentation from 2020 to 2021. The summary lists show that the total number of trips of emergency medical teams without a doctor—PEMS (primary emergency medical service)—and with a doctor—SEMS (specialized emergency medical service)—to the scenes of incidents in this period was 17,446, of which PEMS teams were ordered to the scenes of incidents 13,412 (76.88%) times, while for SEMS teams this figure was 4034 times (23.12%). From the total number of trips for the purposes of this study, a group of road incidents involving single-track vehicles was selected (n = 248; 1.42%). During the analysis, data on the places, dates, and times of the incidents and the type of commissioned team were also included. Patient information included sociodemographic data of the victims, injuries, and clinical statuses. The study was approved by the UPH Bioethics Committee (No. 2/2021). The obtained data were analyzed statistically, and the normality of the distribution was assessed using the Shapiro–Wilk test. In order to calculate the significance of the correlation of the variables, the chi-squared test and Spearman’s Rho test were used. All results were considered significant at *p* < 0.05.

## 3. Results

### 3.1. Characteristics of the Incidents

**Figure 1:**
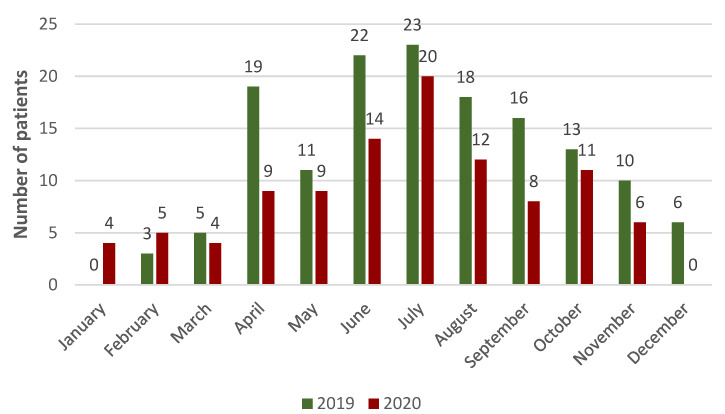
Number of incidents, including months in the audited period of 2019–2020.

The study took into account the number of cases from 2019 (n = 9273; 53.15%) and 2020 (n = 8173; 46.85%). In 2019, there were 146 cases involving single-track vehicles, while in 2020 there were 102 cases (248 in total). A higher intensity of accidents with single-track vehicles was demonstrated in the summer period. A detailed list of interventions in the monthly schedule is presented in Figure 1. During the analyzed period, it was noted that 183 interventions concerned bicycle users (including e-bikes), which is 74% of the total, 61 concerned motorcycle users (25%), and the remaining 4 concerned scooters, including e-scooters (1%). PEMS syndromes were the most common for road incidents involving single-track vehicles, showing a significant correlation between PEMS vs. SEMS (chi-squared test = 59.645; *p* < 0.000). Taking into account the types of incidents in the analyzed period, 62 people were hit, 179 people fell, and 7 people became ill. 

### 3.2. Characteristics of Patients

A total of 41 (16.5%) women and 207 (83.5%) men were injured. The mean age of the victims was 45.66 years (SD ± 20.45). Among the 248 cases, it was specified that 88 (35.48%) of these accidents involved a driver of a single-track vehicle being under the influence of alcohol. There was a significant correlation between gender and driving after alcohol in the study group (Spearman’s Rho = 0.307; *p* < 0.000). Only one woman (2.44% of all the women) in the study group was under the influence of alcohol at the time of the incident, while this applied to as many as 87 men (42.03% of all the men).

### 3.3. Injury to the Body

**Figure 2:**
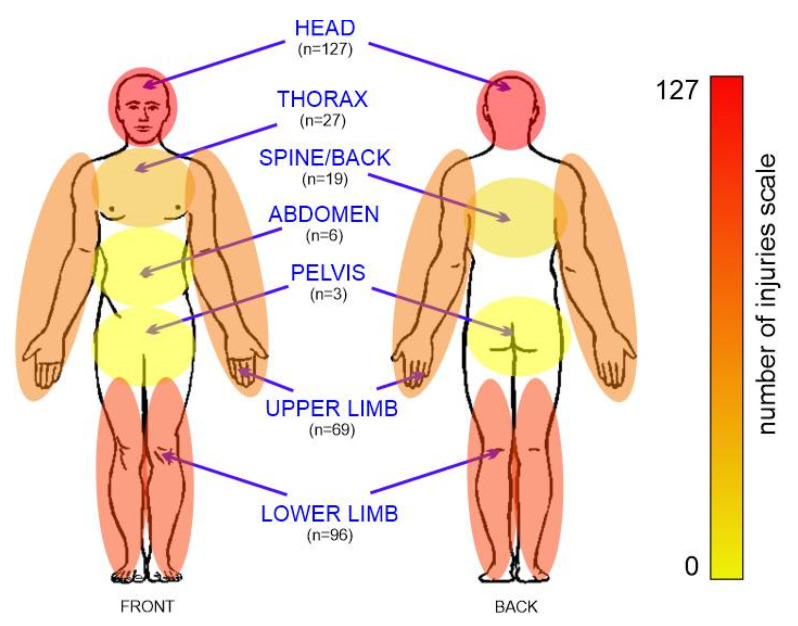
Body injuries of users of single-track vehicles.

During incidents involving single-track vehicles, the head and limbs are the most vulnerable parts of the body to injuries. Figure 2 shows the different types of injuries in a general overview.

The most common injuries suffered by bicycle users were head injuries, which were recorded in 101 (55.19%) of the victims. Lower limb injuries ranked second (n = 60, 32.79%), while upper limb injuries ranked third (n = 46, 25.14%). 

Among motorcyclists, injuries to the lower limbs (n = 35, 57.38%) were the dominant injuries, followed by head injuries (n = 23, 37.70%) and those of the upper limbs (n = 21, 34.42%). Scooter users most often suffered head injuries, as this type of injury was found in 75% (n = 3) of the injured. In second place were injuries of the upper limbs (n = 2.50%), while third place was taken by injuries of the lower limbs and abdomen (n = 1.25%).

**Figure 3:**
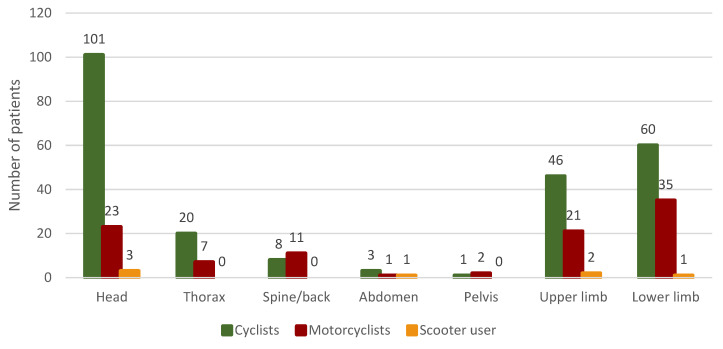
Patients’ injuries, taking into account types of vehicles.

The correlations between the nature of the injuries suffered in individual body regions of the victims and the types of vehicles on which the incidents took place were analyzed. The study showed that there was a significant correlation in the cases of injuries of the spine/back (*p* = 0.001), head (*p* = 0.046), lower limbs (*p* = 0.001), and pelvis (*p* = 0.021). On the other hand, injuries to the abdomen (*p* = 0.125), chest (*p* = 0.931), and upper limbs (*p* = 0.104) showed no significant correlation with types of single-track vehicles. A detailed analysis of patients’ injuries, taking into account types of vehicles, is presented in Figure 3.

**Figure 4:**
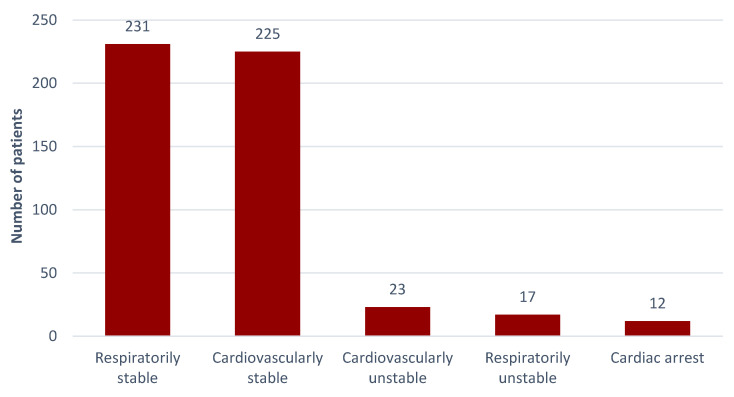
Assessment of the circulatory and respiratory efficiency of the injured.

During the study period, the EMS teams assessed 225 patients as being circulatory-efficient (90.73%), while 23 were inefficient, constituting 9.27% of all of the victims. Efficient breathing was noted in 231 patients (93.15%), and in 17 patients breathing was marked as insufficient (6.85%). Sudden cardiac arrest (SCA) at the scenes of incidents occurred in 12 victims (4.83%), of which 4 withdrew from the medical emergency procedures due to prolonged resuscitation and signs of death. For two patients it was decided to call the Polish Medical Air Rescue (LPR) crew to help the EMS teams at the scene. Detailed datasets are presented in Figure 4.

## 4. Discussion

Traffic accidents are a daily occurrence all over the world. As a consequence of accidents, a huge number of people suffer damage to their health or even death. Due to the widespread use of single-track vehicles as means of transport, users of these vehicles account for a large percentage of the victims of accidents. Data from the report of the European Transport Safety Council (ETSC) from 2019 show that the number of fatalities on roads in Europe amounted to 22,651 people, of which 2909 were fatalities on Polish roads (including 324 people using single-track vehicles).

Despite the presence of a physician in the SEMS teams, who had a greater range of powers and the ability to use more drugs [11] at the scenes of incidents in the analyzed period, PEMS teams were more frequent. In total, PEMS teams were ordered to road incidents involving single-track vehicles 167 times, while this figure was 81 times for SEMS teams (in 2019, PEMS = 101 vs. SEMS = 45; in 2020, PEMS = 66 vs. SEMS = 36). Such a disproportion may result from the fact that in the analyzed period the number of specialist teams has definitely decreased [12]. 

As bicycle users increase in age and speed, they are more prone to developing skull injuries. This may be due to the fact that single-track vehicles are not equipped with structures that protect drivers from hitting another vehicle. A lack of protective clothing also affects the severity and degree of injuries. The medical documentation analyzed during the study did not include the wearing of a helmet, weather conditions, and vehicle speed. Another common problem is that helmet are not fastened properly, which can affect injuries [13]. Following the completion of the study, it was noted that the most common injuries suffered by single-track vehicles were head injuries, which accounted for 36.60% of them (n = 127), while the least common injuries concerned the pelvis (1.20%, n = 3). 

In the analyzed period, the most common body injuries among cyclists were in the areas of the head as well as lower and upper limbs. Lack of a helmet, driving on the road, and no reflectors are just some of the factors that negatively affect road safety. Minimizing these factors will not only have a positive impact on the safety of cyclists and other road users but will also reduce the number of road accidents involving bicycles, which will help maintain health and life [14,15]. Cyclists do not always wear the appropriate protective clothing that motorcyclists do. Adequate head protection can significantly protect the cranial vault, but does not reduce the risk of injuries to other parts of the head (e.g., the mandible) [16].

Lower limb injuries were determined to be the dominant injuries in the cases of motorcyclists, accounting for 57.38% of all injuries, and head injuries took second place. Similar results were presented in the study by Alicioglu et al., in which 212 patients who were hospitalized due to injuries suffered as a result of motorcycle accidents were analyzed. The authors showed injuries to the limbs in 106 victims (50% of all injuries), and head injuries in 103 (48.6% of injuries) [17]. The following factors may have a negative impact on the safety of motorcyclists on the road: no bodywork protection, no seat belts, and no head rests (risk of damage to the cervical spine in an accident) [18]. On the other hand, the positive factors are as follows: the presence of protective clothing, including a properly selected helmet. The lower and upper limbs are the least protected parts of the body among motorcycle users. 

High-energy injuries resulting from road traffic incidents may lead to the development of external or internal hemorrhage as a consequence of hypovolemic shock and hemodynamic failure of victims [19,20]. The types of injuries identified in the study indicate a high risk of multiorgan injuries in these patients [21,22]. Therefore, as part of medical assistance at a scene, it is important to treat each participant of such an accident as potentially traumatized in these areas [23]. To reduce the potential risk factors resulting from injuries, a rapid trauma examination should be performed to look for life-threatening injuries [24,25]. 

The small group of scooter users who were injured indicated the highest percentage of head injuries. The fact that this group of patients was too small means that it does not allow for a reliable assessment of the correlation of variables. The authors note that, as in the case of cyclists, it would be advisable to use protective pads and a helmet for scooter users. 

An additional problem discussed in this study concerns the high percentage of people driving under the influence of alcohol. Among the 248 cases, as many as 88 events (35.48%) were listed in which the driver of a single-track vehicle was under the influence of alcohol. Moreover, significantly more frequent driving after alcohol was found among the injured men (Spearman’s Rho = 0.307, *p* < 0.000).

The COVID-19 pandemic affected many factors of social life and elements of the economy. During its duration, further restrictions were introduced, which apparently resulted in a reduction in road traffic. In 2020, during the pandemic, there were 102 road incidents involving single-track vehicles, while before the pandemic in 2019 there were 146, i.e., 44 (30.14%) less. This indicates a decrease in the number of traffic accidents involving single-track vehicles during the pandemic, which may be due to limitations in the functioning of society. 

**Limitations of the study:** For future research into road accident injuries of single-track vehicle users, a larger sample size study is recommended to investigate this topic in detail. Scooter users accounted for a negligible percentage of the injured, which limits reliable intergroup comparisons. The documentation analyzed retrospectively came only from the ambulance service database. The further fate of hospitalized patients is not known, as is also the case for the results of tests that could have shown additional injuries that were invisible at the scenes of the accidents.

## 5. Conclusions

In the studied group, EMS teams intervened more often in accidents involving men than women. Users of scooters and bicycles most often suffered from head injuries, while motorcyclists suffered from lower limb injuries. Additional solutions should be searched for that would increase road safety and ensure the better protection of single-track vehicle drivers against injuries. It seems to be a good idea to carry out more preventive actions, informing about the dangers of noncompliance with road traffic rules, driving under the influence of alcohol, and the need to properly protect yourself before driving in people who drive single-track vehicles with passengers, such as choosing the right helmet, protective outfit, protective pads, and footwear. 

## Data Availability

Not applicable.
